# A real-time detection and positioning method for small and weak targets using a 1D morphology-based approach in 2D images

**DOI:** 10.1038/lsa.2018.6

**Published:** 2018-05-04

**Authors:** Min-Song Wei, Fei Xing, Zheng You

**Affiliations:** 1 Department of Precision Instrument, Tsinghua University, Beijing 100084, China; 2 State Key Laboratory of Precision Measurement Technology and Instruments, Tsinghua University, Beijing 100084, China; 3 Department of Mechanical Engineering, University of California, Berkeley, CA 94720, USA

**Keywords:** high robustness and accuracy, 1D morphology-based approach, row-by-row structure, real-time detection method, small and weak target detection and positioning

## Abstract

A small and weak target detection method is proposed in this work that outperforms all other methods in terms of real-time capability. It is the first time that two-dimensional (2D) images are processed using only one-dimensional1D structuring elements in a morphology-based approach, enabling the real-time hardware implementation of the whole image processing method. A parallel image readout and processing structure is introduced to achieve an ultra-low latency time on the order of nanoseconds, and a hyper-frame resolution in the time domain can be achieved by combining the row-by-row structure and the electrical rolling shutter technique. Experimental results suggest that the expected target can be successfully detected under various interferences with an accuracy of 0.1 pixels (1*σ*) under the worst sky night test condition and that a centroiding precision of better than 0.03 pixels (1*σ*) can be reached for static tests. The real-time detection method with high robustness and accuracy is attractive for application to all types of real-time small target detection systems, such as medical imaging, infrared surveillance, and target measurement and tracking, where an ultra-high processing speed is required.

## Introduction

Real-time image enhancement and segmentation are critical for weak and small target detection from images with a low signal-to-noise ratio or strong interferences, especially for applications in biomedical image processing^1, 2, 3^, infrared surveillance^4, 5, 6^, and small target measurement and tracking^7, 8, 9, 10^. For example, accurate target recognition and segmentation are crucial for correct diagnosis from an MRI image or proper cell recognition from fluorescence microscope images with uneven background illumination^1, 3^. In a wind tunnel experiment, the use of a small colored tracker to illustrate the fluid field also requires precise pose measurement and an ultra-high tracking speed for images with space brightness uniformity due to high-speed shooting^9^. For target detection by an infrared imaging system or an optical sensor in space, image enhancement has a significant effect on the system or sensor functionality due to the low contrast of the images obtained; in addition, a real-time capability is desired as well^11, 12^. Several different approaches have been used for detection of such small and dim targets, for example, wavelet-based algorithms, inter-frame difference-based algorithms and filter-based algorithms^13, 14, 15, 16^. However, these algorithms cannot be applied to a real-time system because they have a significant latency time due to the complexity of the algorithm; the latency is further worsened by the separation of image readout and image processing because image processing cannot being until the entire image is read out and restored.

Recently, a morphology-based image processing method for image enhancement and segmentation has been developed and was shown to have great potential for small and weak target detection^17, 18, 19^. For an image processed by morphology operations with specific structuring elements (SEs), regions of interest can be effectively enhanced by suppressing noise to a lower level to realize successful target recognition. Nevertheless, approaches employing two-dimensional (2D) SEs in morphology operations still require a large memory space to restore the image, most of which are pixels with no information, leading to a tremendous waste of hardware resources and resulting in a long latency time^20, 21^.

Essentially, a small target usually occupies a connected area, and the connections within such an area can be linked to the pixels in adjacent rows; meanwhile, the image background can be estimated using the local pixels within a row. In this case, a row-by-row (RbR) image processing method can be used estimate the background and determine the connected area for the selected target in a 2D image. A morphology-based one-dimensional (1D) image processing approach for 2D images is introduced in this work. Through 1D SEs in the shape of the line or pair and 1D target recovery and determination, the target can be explicitly separated from the background in a 2D image, and such an image is processed in a parallel structure during pixel readout to realize real-time image processing with significantly reduced hardware resources. The temporal resolution is improved by the use of the electric rolling shutter (ERS) technique. Hardware implementation of such an approach also enhances its real-time performance with an ultra-low latency time at the nanosecond to microsecond level, at least three orders of magnitude better than traditional methods, and the image that must be restored is <1 row of the image instead of the entire image with over one thousand rows. Experimental results indicated that the proposed method can provide highly accurate target positioning. The robustness of this approach was also verified by various tests with different conditions such as interferences of larger objects, intense stray light and an uneven background.

## Materials and methods

### ERS-based parallel pipeline structure for real-time target detection

**Figure 1:**
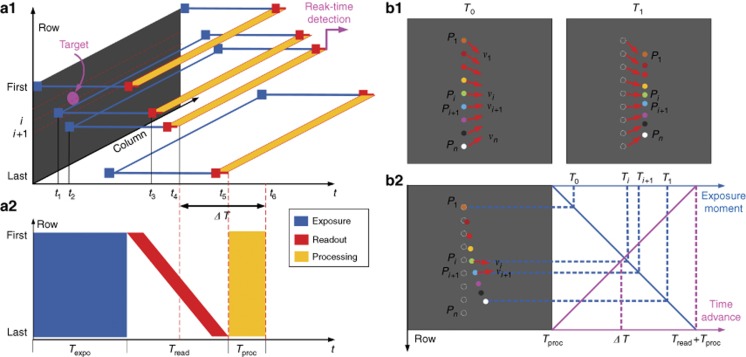
Schematic of image processing of (**a1**) the ERS-based 1D real-time target detection method and (**a2**) traditional methods. Improvement of both the temporal resolution and real-time capability by replacing traditional methods (**b1**) with the proposed real-time method (**b2**). Colorful dots are particles scattering in different rows of a single image, and circles with dashed lines are their original positions at the initial time. Red arrows schematically show the movement of each particle.

Digital image acquisition is composed of image exposure and pixel readout before the image can be processed for target detection, as illustrated in Figure 1a2 for the traditional serial exposure-readout-processing structure^22^. An image is not processed to identify the target until the entire image is restored in this architecture, resulting in a noticeable time delay. The best performance with respect to the processing time, *T*_proc_, of traditional methods ranges from 10 to 100 ms^18, 20, 23, 24^, which is still unacceptable for some particular applications, such as remote sensing or real-time target tracking. Therefore, by processing the image data during pixel readout, an RbR image processing approach is introduced to establish a parallel pipeline structure for real-time 2D image processing, as shown in Figure 1a1.

To fully facilitate the capability of RbR image processing in parallel with the pixel readout, the ERS technique is applied^25^. In Figure 1a1, its exposure and readout mechanism are depicted in blue and red, respectively. The image exposure proceeds in an RbR pattern at a configurable offset d*t*, and the pixels of each row are read out immediately after the current row exposure. Consequently, the pixel exposure-readout between different rows can be independent. For example, the exposure for row *i* begins at *t*_1_, and when the exposure ends at *t*_3_, the pixel value readout for row *i* begins simultaneously, while the pixels in row *i*+1 are still in the exposure stage. By configuring the image sensor carefully, the pixel readout between two images can be seamless, meaning that *t*_5_ is the moment when the pixel readout for the current image ends and the pixel readout for the next image begins. Furthermore, the processing for the pixels in row *i*, as depicted in orange in Figure 1a1, is conducted during the pixel readout in a parallel structure instead of the traditional serial structure, suggesting that the target detection can be fulfilled simultaneously with the completion of row pixel readout at time *t*_4_.

Because of the combination of the ERS technique and the parallel pixel readout-processing structure, real-time target detection is dramatically enhanced, and a hyper-frame resolution in the time domain can be achieved with multiple targets within a single frame of the image. Compared with the global shutter technique shown in Figure 1a2, with which the entire image will be exposed at the same moment and the temporal resolution is limited by the frame rate, *f*_*P*_, ERS enables the information update rate to surpass the limit of the image frame rate because of the exposure time variation among the different rows within a single image, which is imperative for the ultra-fast target tracking system. For instantaneous velocity measurement in fluids, particle image velocimetry is used by taking the images of particles in fluids and analyzing their position variance between different image frames^26^. As shown in Figure 1b1, the colorful dots sketch the particles in the fluids, and by calculating the change in their positions between two consecutive images taken at moments *T*_0_ and *T*_1_, the velocity of each particle can be determined and the property information of the fluids examined. In the global shutter, the information between *T*_0_ and *T*_1_ is always unknown given the frame rate *f*_*P*_, which constrains the information update speed or its temporal resolution d*T* as in Equation (1):


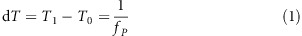


Because of the successive exposure mechanism among the adjacent rows in the ERS mode, the particles in different rows correspond to distinguishable moments in the time domain and can be utilized for temporal resolution enhancement. As shown in Figure 1b2, the uppermost dot *P*_1_ shown in orange is the first particle recorded at moment *T*_0_, which is set to be the initial reference moment for all other particles, with their original positions at that moment shown in circles with dashed lines. As different rows have distinctive exposure moments in the ERS mode, illustrated by the blue line in Figure 1b2, the other particles located below *P*_1_ will be exposed at a subsequent moment and therefore move a certain distance during this period. On the basis of the position variance relative to the reference position and exposure time difference between *T*_*i*_ and *T*_0_, the velocity of particle *P*_*i*_ at moment *T*_*i*_ can be determined (Supplementary Note 1) and reveals the property of the fluids at the inter-frame moment, which could not be obtained through the traditional methods. Assuming that *n* particles, which correspond to different exposure times, are evenly distributed within a single frame of the image, a hyper-frame temporal resolution d*T*_1D_ can be realized using the ERS-based 1D image processing method, as expressed by Equation (2):


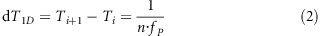


Compared with the resolution in traditional methods given by Equation (1), the temporal resolution d*T* can be improved by a factor of *n*. With the parallel pixel readout-processing structure, the time advance of target detection compared to the traditional serial structure, Δ*T*, is illustrated by the purple line in Figure 1b. We consider two extreme scenarios: (1) when the target is located in the last row of the image, the improvement in the latency time (time from the completion of the target acquisition to the time when the target is identified) is the traditional image processing time, *T*_proc_; (2) when the target is located in the first row of the image, the target will be identified after the readout of the first row, and the latency time improvement is *T*_read_+*T*_proc_. For a target located in an arbitrary row *i*, the reduced latency time Δ*T* is between *T*_proc_ and *T*_read_+*T*_proc_, as explicitly illustrated in Figure 1a. The parallel structure-based 1D image processing method with a hyper-frame temporal resolution is attractive for high-speed time-critical applications such as medical imaging, target tracking and measurement and computer-vision assisted robotics^27^. For example, a remote sensing satellite requires an ultra-high processing and update speed for attitude determination from optical sensors, given that the distance mismatch would be 7.1 m for the latency time of 1 ms.

### One-dimensional morphology-based method for selective target detection

**Figure 2:**
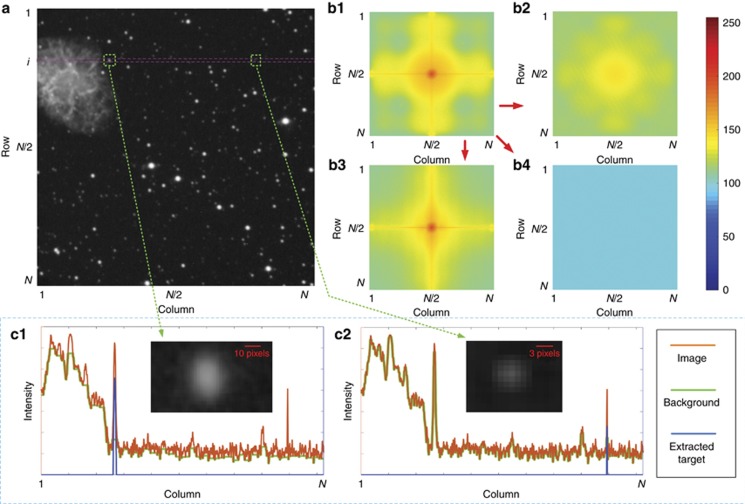
Image properties in the spatial domain and the frequency domain: (**a**) original image with stars of different sizes, as well as a nebula and a galaxy; FFT results of (**b1**) the original image, (**b2**) single pixel noise, (**b3**) background noise and (**b4**) targets. Centers and edges of the FFT results correspond to lower and higher frequencies, respectively. Illustration of RbR image processing for targets in row *i* with different sizes: (**c1**) extraction of a target with a larger size and (**c2**) extraction of a target with a smaller size. Insets are the zoom-in view of the extracted targets in **a**.

Typical images contain small targets surrounded by irregular backgrounds in a large area, such as stars (target) with a nebula or a galaxy (interference or background), as shown in Figure 2a. A point spread function (PSF), expressed by Equation (3), can be employed to approximate a small target^13, 28, 29^, which is basically a connected area with a decrease in brightness from the target center to its edge, as expressed by a Gaussian distribution (Supplementary Note 2 and Supplementary Fig. S1).


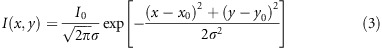


where *I*_0_ is the total energy pertaining to the target, (*x*_0_, *y*_0_) is the expected center of the target and *σ* is the Gaussian radius, which is related to the concentration of the PSF energy distribution.

There are different types of noise and interference within an image containing the desired small target, and their spatial scales are chosen as the criterion for classification. If the desired target that spreads from several pixels to tens of pixels in size with different shapes has the feature scale of *K* in the spatial domain, the feature sizes of other signals in the image can be classified as larger than *K*, *K*_*l*_, and as smaller than *K*, *K*_*s*_. The image background, large scale noise such as fixed pattern noise comparable to the whole image in spatial scale, and large-scale interferences are all treated as background noise with feature *K*_*l*_, whereas smaller undesired objects and single pixel noise such as hot pixel noise with feature *K*_*s*_ has a smaller scale than the desired target. Figure 2b explicitly shows the composition of an image with *N* rows and *N* columns in the frequency domain. The center and edge correspond to the lower frequency and higher frequency, respectively, and the intensity is the logarithmic value of the fast Fourier transform (FFT). When performing the FFT on a 2D image, the 1D FFT of each row is taken to form an intermediate image, followed by the 1D FFT of each column in the intermediate image. The FFT results of Figure 2a is shown in Figure 2b1, and it is obvious that the desired target signal is overwhelmed by the background noise, whose FFT result, shown in Figure 2b3, features dominant low frequency components due to the continuous distribution of the background noise signal with size *K*_*l*_. For the noise with size *K*_*s*_, the FFT result, shown in Figure 2b4, is tremendously lower in the low-frequency domain compared with the background signal and exhibits a uniform distribution throughout all frequencies, in accordance with its random and isolated distribution in the spatial domain. After filtering all undesired parts from the original image, the FFT result of the desired targets with size *K* is plotted in Figure 2b2 with small components ranging from low frequency to intermediate frequency. The 2D component classification, which is based on the properties in the spatial domain, can also be applied to 1D components (Supplementary Note 3).

Image processing was achieved by a mathematical morphology-based image processing approach. Erosion and dilation are two basic operations in mathematical morphology^30, 31^, and they are expressed by Equations (4) and (5), respectively.


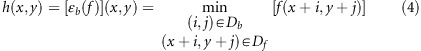



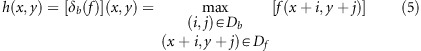


where *f*(*x*, *y*) and *h*(*x*, *y*) are sets to represent the gray value (energy intensity) of images *f* and *h* at a certain point (*x*, *y*), respectively. *b* is the SE, *D*_*b*_ is the domain of *b*, and *D*_*f*_ is the domain of *f*.

The traditional SEs utilized for 2D image processing are 2D shapes such as a rectangle or an ellipse^30^. However, extra memory space is required to restore several rows of an image, in accordance with the applied 2D SEs in the column direction, and most of the restored pixels are merely background noise, leading to inefficient usage of the hardware resources, as well as a greater latency time. As 1D SEs, including a line and a pair, fit well in the pipeline structure of image readout, as shown in Figure 1a, they are employed in the 1D image processing method, and thus, the image is processed row by row by the 1D morphology-based image processing approach, as shown in Figure 2c. The relationship between the length (*L*) of the SEs employed in the 1D image processing method and the feature scale (*K*) of the components in an image can be expressed by Equations (6) and (7):


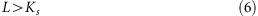



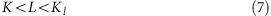


Erosion can be used to remove the single-pixel noise or smaller objects in the image with Equation (6), while through a combination of erosion and dilation, background noise can also be obtained for target enhancement using Equation (7). Taking the row *i* in Figure 2a as an example, there are two typical targets with lengths of 25 pixels (*K*_1_=25) and 7 pixels (*K*_2_=7), as shown in the insets of Figure 2c, and part of a nebula as interference (*K*_*l*_). Assuming 1<*L*<*K*_2_, a combination of erosion and dilation can remove both the nebula and the larger target as the background noise, leaving the smaller target only as the image enhancement result shown in Figure 2c2, while if *K*_1_<*L*<*K*_*l*_ is satisfied for the image enhancement, followed by another erosion with *L*>*K*_2_, only the larger target is extracted, as shown in Figure 2c1. By customizing the parameter *L*, targets with various sizes can be detected selectively.

### One-dimensional processing procedure for weak and small target detection and positioning

**Figure 3:**
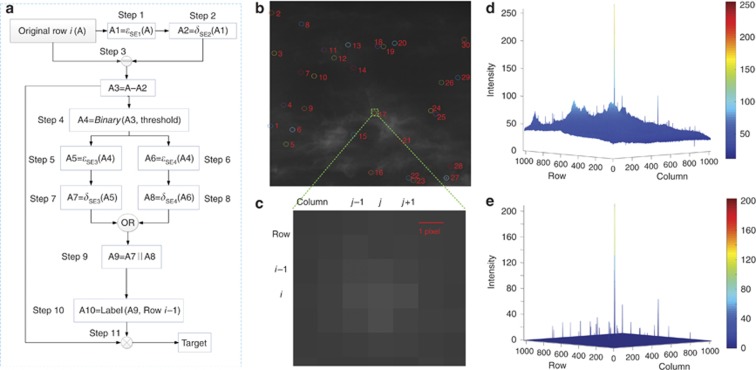
(**a**) Flow chart of the 1D morphology-based image processing approach for small and weak target detection and positioning. (**b**) Original image and its final processing result with 30 identified targets. (**c**) Zoom-in view of a specific target in **b**. Intensity distribution of (**d**) the original image and (**e**) identified targets.

Figure 3a details the procedures of the 1D morphology-based image processing approach for weak and small target detection and positioning. The connection of a target given by Equation (1) can always be linked between the adjacent pixels in successive rows. Figure 3b illustrates an image with 30 desired targets surrounded by observed noise and intense background interference. The zoom-in view of one of the small and weak targets ~3 × 3 pixels in size is shown in Figure 3c. The challenge for processing the image is to identify the weak and small target with strong background noise or a low signal-to-noise ratio^32, 33^. Image enhancement is implemented by precisely separating the background noise from the original image (Figure 3d), and the background noise (Supplementary Fig. S3a) is determined by image erosion followed by dilation as steps 1 and 2 in Figure 3a. The SEs for both steps are line elements in the same direction as the pixels in a row. The erosion with a line SE can remove the positive signals with a small scale, including the target, whereas the dilation can eliminate the negative noise, such as a dark point due to a broken pixel of the image sensor. Image enhancement is achieved by subtracting the background from the original image as step 3, and then, the enhanced targets can be separated from the background with the employment of a threshold as step 4 (Supplementary Fig. S3b and S3c). Further noise reduction for false positive targets, as well as for single pixel noise, is conducted through the erosion with pair SEs in both the horizontal and vertical directions as steps 5 and 6 (Supplementary Note 4).

However, the erosion in two orthogonal directions also removes part of the extracted target, undermining the accuracy of further target positioning. Hence, immediate dilation by the same SE after erosion is imperative as the target recovery method, steps 7–9 in Figure 3a. The target recovery keeps the targets intact, as shown in Supplementary Fig. S4, and is crucial for small and weak target positioning. Simulation results suggest that such a target recovery can enhance the target positioning accuracy by a factor of 3 (Supplementary Note 4 and Supplementary Fig. S5).

Image enhancement and target extraction have been achieved in an RbR pattern after step 9 in Figure 3a, with the target separated in adjacent rows. As the subsequent pixel is unknown for the current pixel in the as-proposed 1D image progressing approach, a row-based connection strategy is introduced as step 10 in Figure 3a. Considering the PSF properties of a small target in Equation (3), the component for a certain target is determined pixel by pixel, and the pixels with the same connected-component label number constitute a specific target for which the centroid position can be calculated by using all pixels with the same label number. The method to determine the label number of a certain pixel is given in Equation (8) based on a priori information of the current row and one previous row:


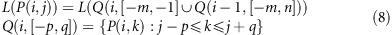


where *L*(*P*(*i*,*j*)) is the number of the connected-component label for pixel *P*(*i*,*j*) and *Q*(*i*,[−*p,q*]) is a set of pixels in row *i* from column (*j*−*p*) to (*j*+q). The label number for the pixel *P*(*i*, *j*) is predicated on the label number of nearby pixels defined by *Q*. The parameters *m* and *n* are adjustable based on the target properties, such as the size and signal completeness, which can be abstracted as a function of the energy distribution of a target. For the target in Figure 3c, it is practical to have *m*=1 and *n*=1 since the target has a relatively standard Gaussian distribution with a significantly small size. In this case, *P*(*i*, *j*) is determined by four pixels, including three in the previous row, which are *P*(*i*−1, *j*−1), *P*(*i*−1, *j*) and *P*(*i*−1, *j*+1), and one pixel just prior to it in the same row, *P*(*i*, *j*−1). After the labeling determination, all valid pixels of a continuous target are connected with the same label number for centroid positioning. Figure 3b shows the final processing results with 30 detected targets, and their intensity distribution is illustrated in Figure 3e.

## Results and discussion

### Real-time image processing implementation

Generally, the pixels containing the information for the small and weak targets only account for <1% of the total pixels in an image to be processed. The other more than 99% ‘dumb’ pixels only serve as the image background, and it would be a tremendous waste of resources to restore the entire image. Meanwhile, since the row image data are processed by the 1D image processing method during its readout based on an RbR pattern, it is unnecessary to restore the entire image, significantly reducing the memory space for image storage. Theoretically, just one more row of currently processed image data is required to be restored in order to implement the target segmentation.

By using the 1D morphology-based image processing method, the image has to be processed row by row during the data readout, and thus, the proposed approach is accomplished by logic circuits in the hardware, instead of codes in the software^34^. For the hardware implementation, the operation of erosion or dilation can be abstracted as a minimum or maximum of a dynamic array specified by SEs. For example, to realize step 1 in Figure 3a, a first-in, first-out (FIFO) buffer with a depth equivalent to the length of the line SE is created to function as the dynamic array, and the minimum among all the data in this buffer will be output every time the buffer is updated with new readout data. It is noticeable that for the pair SE specifying two adjacent pixels in the vertical direction of an image, a buffer with the width of a whole row is allocated since the two pixels are in two rows. The shape of each SE employed in the image processing approach and the required storage space for the corresponding FIFO buffers, as well as the latency time, are summarized in Supplementary Table S1 (Supplementary Note 5). The total storage space required for this 1D image processing method is determined by the total depth of all the buffers and the necessary space for the original image to realize the synchronization, which is less than 1 row for the entire image with 1024 × 1024 pixels in 8 bits, or 0.1% of the whole image. The total latency time is calculated as the readout time for several tens of pixels, which is 2–3 μs with a typical 25 MHz readout clock. Target detection could thus be implemented and output within several microseconds after the time at which the last row of the target is acquired through pixel readout (*t*_4_ in Figure 1a1) regardless of the completion of the whole image readout. With a higher-speed readout clock at several GHz, the latency time could be further reduced by two or three orders of magnitude to several nanoseconds. Assuming target detection is not fulfilled until the completion of the whole image readout (equivalent to the target being present in the last row), the minimum processing time with the state-of-the-art real-time image processing methods is several tens of milliseconds^18, 24^ (*T*_proc_>10 ms), which could be improved by a factor of at least 5000 with the proposed real-time method. Furthermore, if the target is located in the very first row of the image, there would be an additional several tens of milliseconds of time advance in target detection given the redundant waiting time for pixel readout (*T*_read_≈42 ms with a megapixel image sensor and a 25 MHz readout clock) before image processing in the traditional image readout-processing series structure in Figure 1a2.

### Centroiding precision for static small target determination

**Figure 4:**
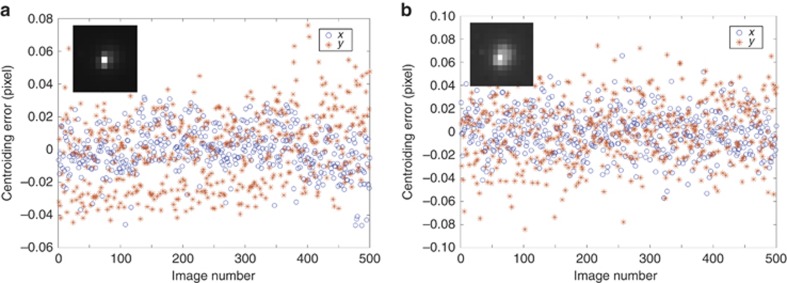
Centroiding precision under static tests with different target sizes. Test results with the target size of **(a**) 3 × 3 pixels and (**b**) 7 × 7 pixels. Insets are typical target spots for each test.

Simulations have been carried out to optimize the parameters and structure of the approach for small target detection (Supplementary Note 6). Static images with a single target of different sizes were employed for the method performance test to evaluate the precision of the target centroid positioning, which is essential for the accuracy of the target tracking system. The single target is simulated by a collimator and tuned by adjusting the exposure time of the CMOS image sensor to two different sizes: 3 × 3 pixels and 7 × 7 pixels. A total of 500 images were obtained for each precision estimation test, with rows and columns in the image defined as the *x* and *y* directions, respectively. The test results shown in Figure 4 indicate that the centroiding precision for the smaller spot is 0.013 and 0.023 pixel (1*σ*) in the *x* and *y* directions, respectively, and increases to 0.019 and 0.027 pixel (1*σ*) for the larger spot. The accuracy enhancement of the centroiding position with the smaller target can be attributed to the focused energy distribution of the target and the relatively less noise in the background.

### Method robustness experiments

**Figure 5:**
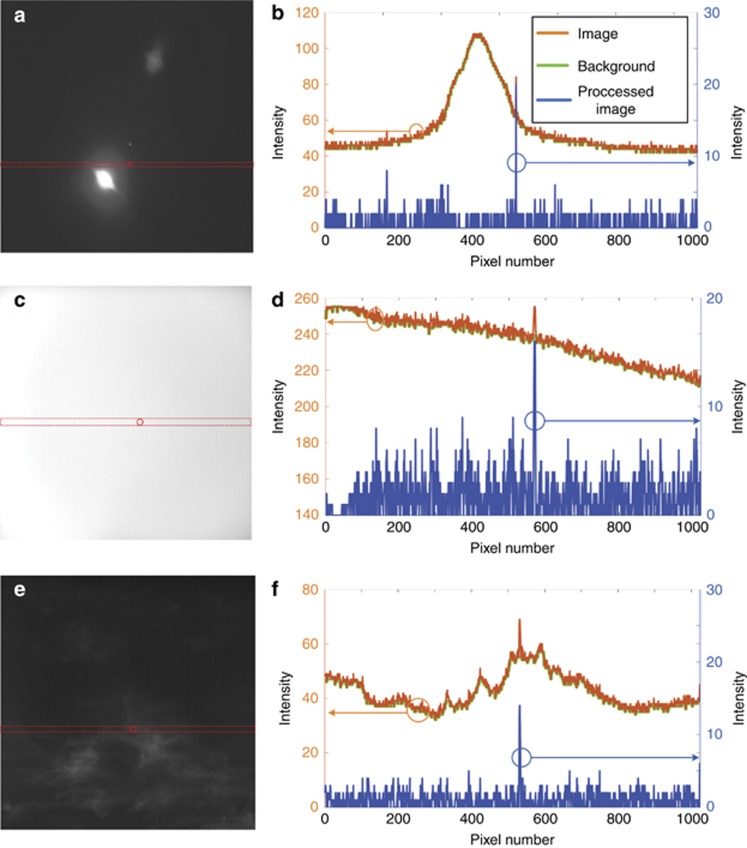
Images with different background conditions and interferences: (**a**) moon, (**c**) stray sunlight and (**e**) clouds. The corresponding processing results of rows designated with red dashed lines obtained by the 1D morphology-based approach: (**b**) target at the edge of the moon, (**d**) target immersed in the stray sunlight and (**f**) target surrounded by clouds. The legend in **b** could also be applied in **d** and **f**.

For the robustness test, the 1D image processing method was applied to images with different background conditions and interferences, such as non-target objects with larger size (moon in Figure 5a), negative noise (Supplementary Fig. S8a), strong stray light (sunlight in Figure 5c) and large-scale clutter (clouds in Figure 5e). The two false positive targets in Figure 5a are the moon and its reflection inside the baffle. The intensity of the moon center is saturated (255 in terms of the gray value), as shown in Supplementary Fig. S8d, and the gray value of the moon edge gradually decays to ~50, as shown in Figure 5b. The negative noise is a dark point spreading to a space of 3 × 3 pixels, with its intensity much lower than that of the background in Supplementary Fig. S8b. In Figure 5c, it is obvious that the left side of the image is severely affected by the stray light from the sun and sky background, with part of the image even saturated. The stray light introduces an intense background noise above 210 in gray value with a gradation, while the clouds in Figure 5e lead to an uneven background with the gray value varying from 30 to 60, approximately. After the background analysis, all undesired targets (negative noise and the moon) can be effectively removed, as shown in Supplementary Fig. S8b, and S8d and Figure 5b. The interferences (sunlight, clouds) can also be significantly suppressed or filtered out, as shown in Figure 5d and 5f. Figure 5b, 5d and 5f, demonstrates that the image can be enhanced for target extraction (blue) after the background (green) is removed from the original image (orange). The desired targets circled in red in Figure 5a, 5c and 5e, are at the edge of the moon, immersed in the stray sunlight and surrounded by the clouds, respectively. By using the 1D image processing method, they can be identified and are indicated by the blue circles in Figure 5b, 5d and 5f, with maximum gray values <20, significantly weaker than the intensity of the background and interferences. The test results indicate that the proposed method exhibits excellent robustness to harsh working conditions in different application scenarios.

### Method accuracy analysis for small targets with interference

**Figure 6:**
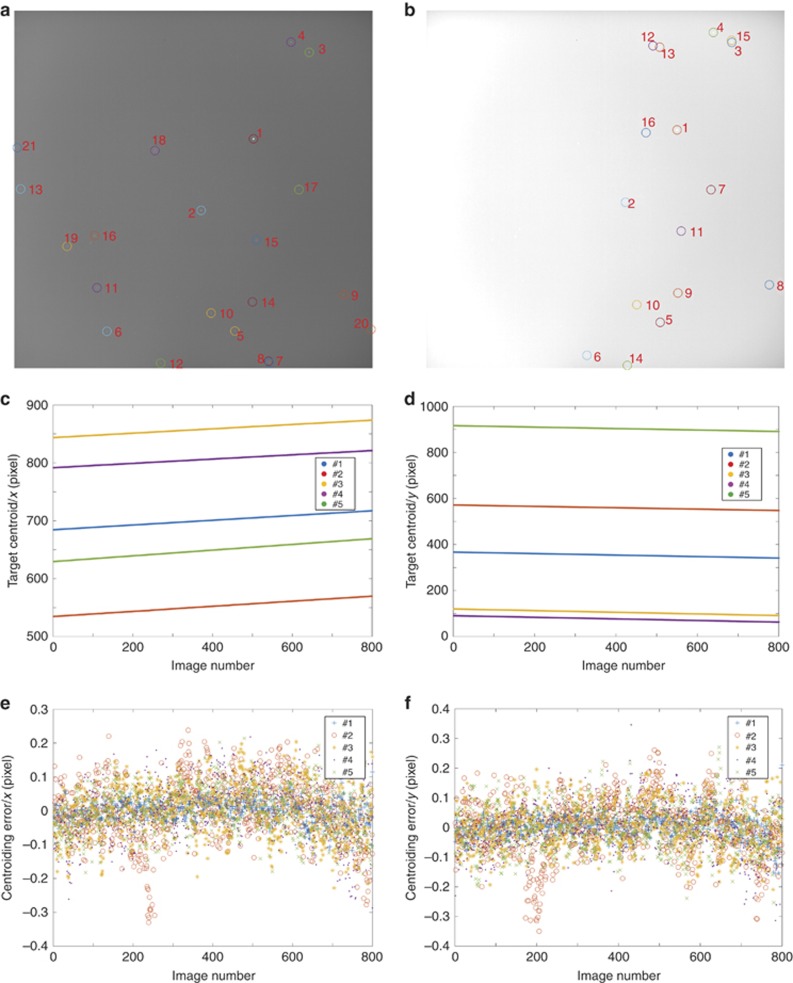
Method accuracy analysis on images with intense interference from stray light. (**a**) First and (**b**) last images during a period before sunrise with the target recognition results. Centroid positions of five targets during this period in the (**c**) *x* direction and (**d**) *y* direction, and their positioning errors between calculated results and fitting results in the (**e**) *x* direction and (**f**) *y* direction.

As the 1D morphology-based approach shows good robustness, its accuracy under interference is examined by fixing a star tracker prototype onto a tripod in a field experiment, as shown in Supplementary Fig. S9a. For method accuracy estimation under the interference of sunlight, 800 images taken by the prototype before sunrise were analyzed, with the average brightness ranging from <120 to ~240 for the saturation brightness value of 255, as shown in Supplementary Fig. S9b. All images are processed with the proposed approach, and the target recognition results of the first and last images are shown in Figure 6a and 6b, respectively. The total test time for recording all the 800 images is ~3 min, and during this period, all extracted targets in the first image remain in the field of view of the star tracker until the end of the test, which could also be verified by comparing the target distribution between Figure 6a and 6b. The centroid information of the five targets with the identification probability of 100% during the test is shown in Figure 6c and 6d. The fitting curves of their centroid positions in the *x* or *y* direction serve as the benchmark for the position accuracy estimation, and the target positioning errors are shown in Figure 6e and 6f, for the *x* and *y* directions, respectively. Table 1 summarizes the test results, which suggest that the proposed method has an accuracy of better than 0.1 pixels (1*σ*). Compared with the static test results, the reduced accuracy in the field experiment could be attributed to environmental factors such as the wind or clouds during the field experiment, which will undermine the benchmark for the accuracy estimation.

## Conclusions

A real-time image processing approach is introduced for small and weak target detection in this work. Mathematical morphology operations with 1D structuring elements have been employed for 2D image enhancement and selective target detection. The 1D image processing approach is embedded into hardware to process the image in the parallel processing structure row by row during the data readout. The breakthrough in hyper-frame temporal resolution is achieved by also using the ERS technique. Small and weak target recovery and determination are studied for positioning accuracy. A case study verified that the 1D morphology-based method shows good robustness in different working conditions. Furthermore, lab test and field experiment results suggested that the proposed method can effectively realize target positioning with an accuracy of better than 0.1 pixels (1*σ*). The total latency time of such an approach is <3 μs with a 25 MHz readout clock, which remains almost constant with an increase in the image spatial resolution, especially in the number of rows. With the merits of real-time performance, high accuracy and robustness, the proposed image processing method is promising for applications in biomedical image processing, infrared surveillance and target measurement and tracking.

## Figures and Tables

**Table 1:** Sky night accuracy test results

Target No.	1	2	3	4	5
Accuracy in the *x* direction (1*σ*)/pixel	0.03	0.09	0.08	0.08	0.06
Accuracy in the *y* direction (1*σ*)/pixel	0.04	0.10	0.07	0.08	0.08
