# Supplementary material for: A real-time detection and positioning method for small and weak targets using a 1D morphology-based approach in 2D images
Source: Light Sci Appl. 2018 May 4;7:18006–. doi: 10.1038/lsa.2018.6 (PMC6060073; doi:10.1038/lsa.2018.6)
Supplement: Supplementary Information [file lsa20186x1.pdf]

Supplementary information

A real-time detection and positioning method for small and weak targets using a 1D morphology-based approach in 2D images

Minsong Wei[1,2,3], Fei Xing[1,2,*] and Zheng You[1,2,*]

1. Department of Precision Instrument, Tsinghua University, Beijing 100084, China
2. State Key Laboratory of Precision Measurement Technology and Instruments, Tsinghua University, Beijing 100084, China
3. Department of Mechanical Engineering, University of California, Berkeley, Berkeley, CA 94720, USA

*Corresponding author：

Fei Xing

Telephone: +86-10-62776000

Fax: +86-10-62781981

Email: xingfei@mail.tsinghua.edu.cn

Zheng You

Telephone: +86-10-62782308

Fax: +86-10-62781981

Email: yz-dpi@mail.tsinghua.edu.cn

Note 1. ERS based velocity determination in PIV

Assuming that (x_i, y_i) and (x_0, y_0) are centroid positions of particle P_i at the moment T_i and T_0, respectively, as shown in Figure 1(b2), the velocity of particle P_i at the moment T_i could be determined by Equations (S1) and (S2):

$$v_{ix} = \frac{x_i - x_0}{T_i - T_0} \quad \text{(S1)}$$

$$v_{iy} = \frac{y_i - y_0}{T_i - T_0} \quad \text{(S2)}$$

where v_{ix} and v_{iy} are the velocity of P_i at the moment T_i in horizontal direction and vertical direction, respectively.

Note 2. Small target simulation based on PSF model

A two-dimentional Gaussian distribution model based on Equation (3) is employed for a target spot simulation and the simulated target has a typical size of 5×5 pixels without background noise, as shown in Figure S1.

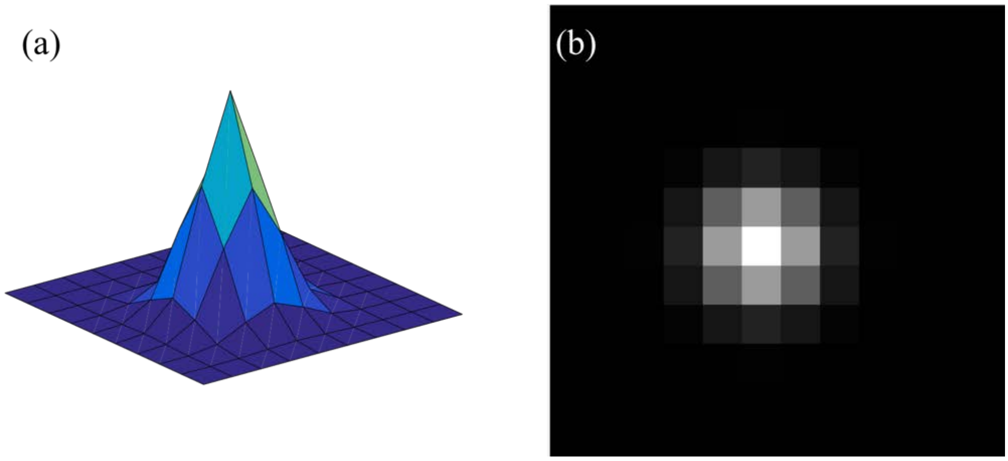

Figure S1 A simulated target with a typical size of 5×5 pixels and no background noise: (a) energy distribution; (b) the simulated image.

Note 3. FFT results of 1D targets with different sizes

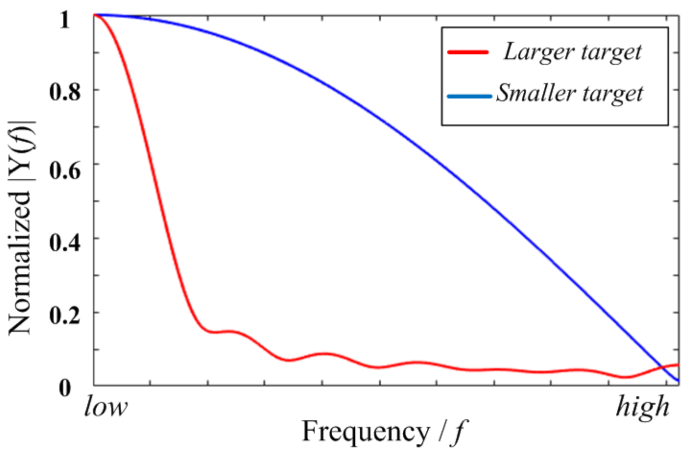

Figure S2 FFT results of 1D targets with different sizes.

When performing FFT on 1D objects, with the decrease in target scale, the spectrum in the frequency domain expands from low frequency to high frequency, which is consistent with the FFT results on 2D objects. As shown in the Figure S2, a sharp peak at the low frequency corresponds to a larger scale 1D target (Figure 2(c1)) while a broader distribution over a wide frequency range corresponds to a smaller scale 1D target (Figure 2(c2)).

Note 4. Illustration of the 1D image processing approach with target recovery

After a threshold is applied to fulfil the image segmentation with all pixels brighter than the threshold value labeled as 1 and others labeled as 0, the erosion of the binarized image by a pair SE in both *x* and *y* directions could further remove single-point noise effectively. Targets with more than one valid pixel connected to each other could survive after the erosion. By comparing the results with (Figures 3(b) and (e)) or without erosion (Figures S3(b) and (c)), more than 90 false targets have been eliminated after the erosion. Since part of the targets could also be eroded by aforementioned de-noise processing, the target recovery method is introduced in Figure S4. After the OR-

operation of two images processed by the erosion-dilation in two directions, the target keeps intact while single-point noise could be removed.

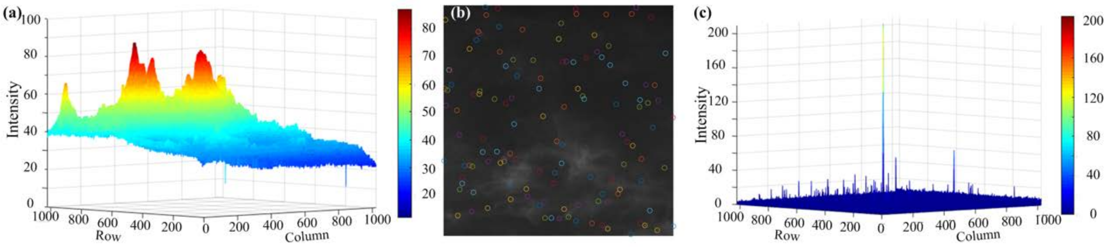

Figure S3 (a) The intensity distribution of determined background. (b) The target extraction result without erosion for single-point noise removal and (c) intensity distribution after step 4 in the 1D morphology based method.

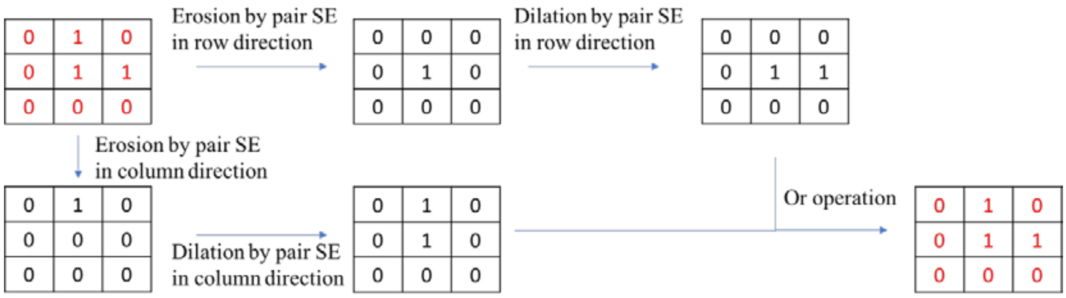

Figure S4 Target recovery implementation by dilation and Or-operation.

Figure S5 shows the centroiding errors of target determination with and without the target recovery. A typical Gaussian noise with a mean value of 5 and a standard deviation of 0.1 has been added to the simulated image. It is obvious that the calculated RMS centroiding errors with and without target recovery are approximately 0.02 pixel and 0.07 pixel, respectively. The target centroid accuracy is enhanced by the additive dilations.

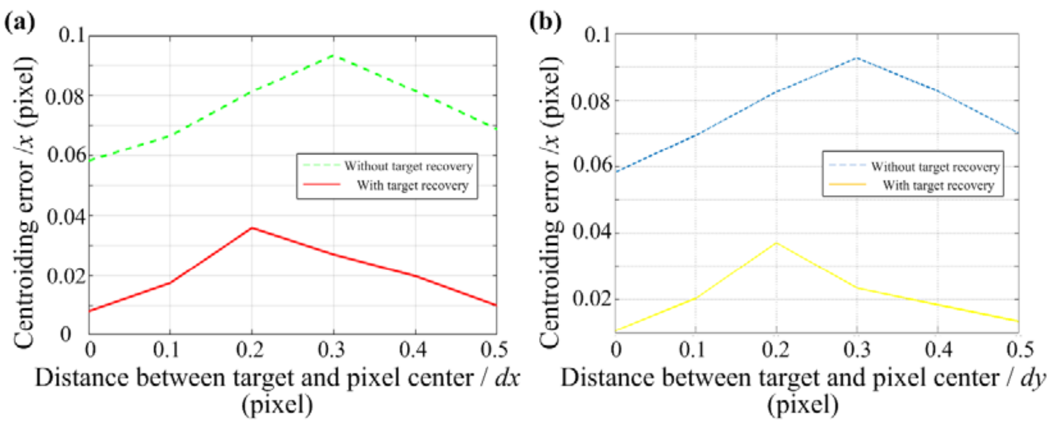

Figure S5 Target centroiding errors in (a) *x* direction and (b) *y* direction with and without target recovery.

Note 5. Table S1 Storage space and latency time for method hardware implementation

Processing Step	Buffer space / buffer depth × bits#	Latency time / pixel readout time
1: Erosion by SE1 with line length L_{SE1}	L_{SE1} ×8	L_{SE1}/2 or (L_{SE1} +1)/2, whichever is an integer
2: Dilation by SE2 with line length L_{SE2}	L_{SE2} ×8	L_{SE2}/2 or (L_{SE2} +1)/2, whichever is an integer
3: Subtraction	0	0
4: Image binarization	0	0
5: Erosion by pair SE in row direction	2×1	2
6: Erosion by pair SE in column direction	*Lrow*×1	2
7: Dilation by pair SE in row direction	2*1	2

8: Dilation by pair SE in column direction	*Lrow*×1	2
9: OR operation	0	0
10: Labeling	*Lrow*×1	1
11: Results output	0	1

#For pixel output in 8 bits, storage space is expressed as depth×8 bits; for data after binarization, buffer depth is expressed as depth×1 bits.

Note 6. Line length optimization for SE

Step 1 and step 2 in Figure 3 are background analysis for image enhancement. Since the background is subtracted from the original image in step 3, less useful information left in the background will contribute to a more accurate target segmentation and target centroid calculation. Simulations are conducted with the target center locates 0.3 pixel and 0.2 pixel away from the pixel center in *x* direction and *y* direction, respectively (*dx*=0.3 pixel, *dy*=0.2 pixel). The systematic error is corrected in the simulation results and the centroiding error induced by the line length of SE1 in step 1 with respect to the size of simulated target is illustrated in Figure S6.

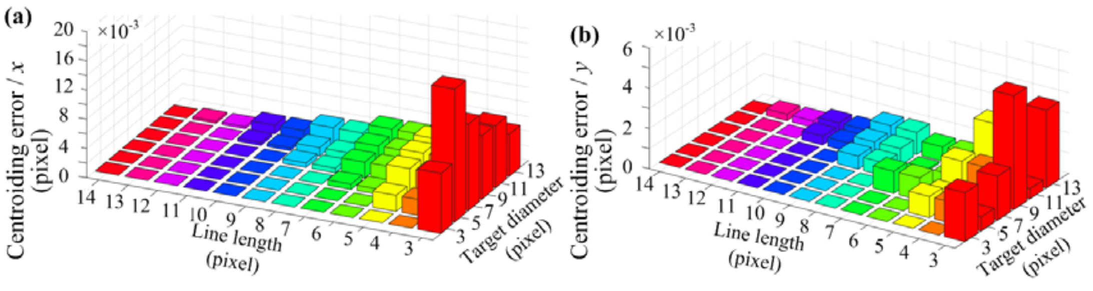

Figure S6 Optimization of SE line length in step 1.

Since the erosion in step 1 happens in the x direction, it has a more significant influence on the target centroiding accuracy in x direction than that in y direction. It is noted that there will be no centroiding error in both x and y directions, if the line length of SE1 is larger than the diameter of the target spot. In other word, since the goal of the erosion in step 1 is to eliminate all the target information for a more accuracy background analysis, a SE with the line length larger than the target size could achieve the goal by removing the whole target and inducing no error when further subtracting the background from the original image. Similar to that, the positive target whose size in x direction is smaller than the line length of SE will be removed by the erosion, whereas the negative noise, such as a broken point on the CMOS image sensor whose size in x direction is smaller than the line length of SE will be removed by the dilation in step 2. However, in order to remove a positive object with the size much larger than the line length of SE utilized in erosion, such as the moon, the line length of SE for dilation should not be smaller than that for erosion. Figure S7 illustrates the image processing results with different line length (L) of the SEs in steps 1 and 2. With a smaller L, the bigger and brighter non-target object (the moon) could be completely removed since the combination of the erosion and dilation could keep such object intact as background which will be subtracted from the original image, while its edge could not be removed effectively with a bigger L. However, some desired small targets could not be recognized when L is too small because any target with the scale smaller than L will be eliminated during erosion and the number of extracted targets will decrease with smaller L until L is smaller than any desired targets.

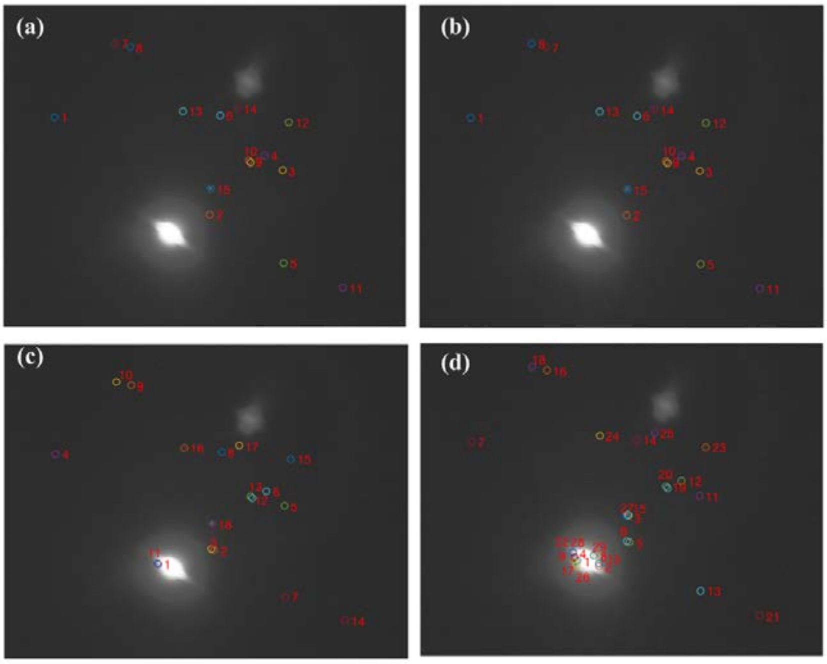

Figure S7 Image processing results of star images with interference of the moon by using SE with different line length (*L*) (a) *L* =5; (b) *L* =7; (c) *L* =9; (d) *L* =11.

Note 7. Method robustness experiments

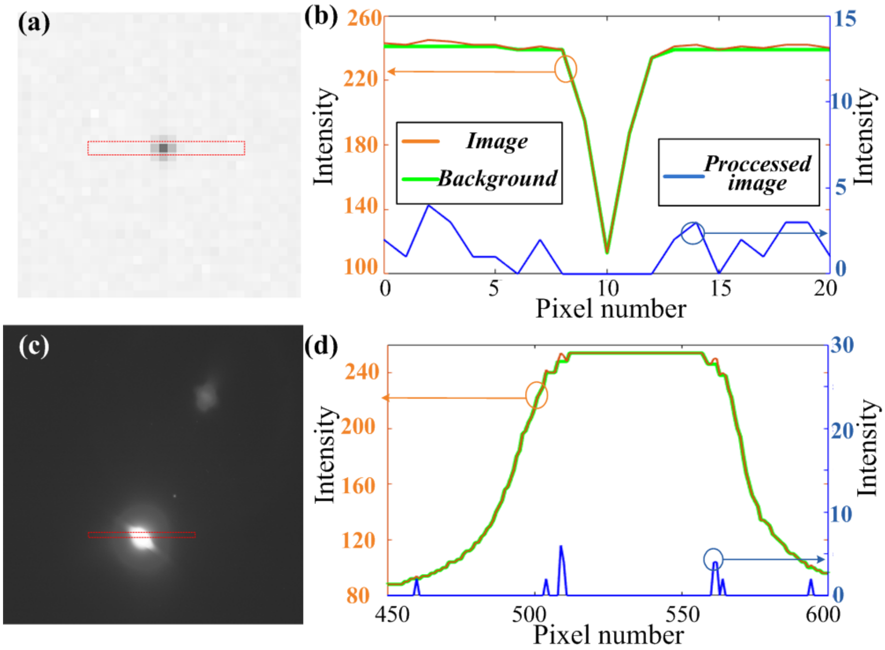

Figure S8 Images with different interferences and their removal by the 1D morphology based approach: (a, b) negative noise, and (c, d) moon. The legend in (b) could also be applied in (d).

Note 8. The sky night field test

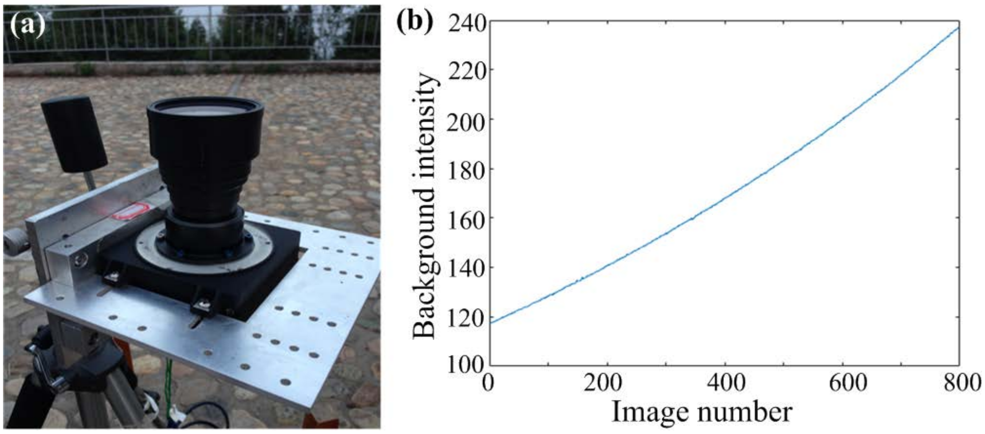

Figure S9 (a) The experiment setup for the sky night field test and (b) background intensity of images obtained before sunrise.
